# Breaking Trust: How Covid‐19 Communication Failures Undermined Public Confidence in US Health Officials—A Narrative Review

**DOI:** 10.1002/puh2.70373

**Published:** 2026-09-15

**Authors:** Matthew L. Harrison

**Affiliations:** ^1^ Department of Emergency Medicine The University of Texas Southwestern Medical Center Dallas Texas USA

**Keywords:** coronavirus disease 2019 (Covid‐19), distrust, politics, polling, public health, public trust, quarantine, severe acute respiratory syndrome coronavirus 2 (SARS‐CoV‐2), timeline, vaccination policy

## Abstract

National polling shows US citizens increasingly distrust those tasked with making our public health decisions. This dilemma appears to have been driven in part by evolving policy recommendations, communications from health officials and medical agencies that were confusing to some, and seemingly hypocritical behavior among US political leaders during the Covid‐19 pandemic. The following review provides a chronological timeline of multiple pandemic‐era events as well as communication and behavior missteps potentially responsible for the growing public distrust. It also suggests possible solutions to ameliorate the problem going forward.

## 1 Introduction

Polling data from the United States indicate that public confidence in health officials, governmental health agencies, and elected leaders responsible for healthcare policy has declined significantly since the onset of the Covid‐19 pandemic. A considerable proportion of the American populace now express skepticism toward these institutions, politicians, and health leaders. Inconsistent public health communication and perceived contradictions in leadership behavior are frequently cited as primary contributors to this erosion of trust [1, 2, 3]. Therefore, I sought to provide a narrative timeline of the major events potentially responsible for the public's growing distrust.

## 2 Methods

Multiple academic and general search sources were utilized to investigate important events, policy directives, executive orders, governmental agency announcements, and studies related to health communication that took place during the pandemic. These include PubMed/Medline, Scopus, Web of Science, Google, Google Scholar, White House Press Briefings, the Associated Press, Cochrane Library, the David J. Spencer CDC Museum, and Dr. Tom Frieden's Blog. Search terms included “Covid,” “Covid‐19,” “SARS‐CoV‐2,” “Covid‐19 vaccine,” “Covid‐19 timeline,” “Covid‐19 major events,” “Covid communication,” “Spikevax,” “Comirnaty,” “Janssen COVID‐19,” “Public Opinion,” “Anthony Fauci,” “Donald Trump,” “Joe Biden,” “Rachel Walensky,” and combinations of the above terms. Next, to minimize omission bias and ensure that no relevant studies or information were overlooked, ChatGPT, Google Gemini, and Perplexity were independently tasked with generating timelines of major Covid‐19–era events and identifying supporting sources. Findings from this supplementary search were compared with those from the conventional academic literature search to assess the completeness of the initial search.

Academic and general search engines were also utilized to evaluate public polling and social media trends shortly after each event. It was determined that serial public opinion polling and social media trends, observed in the aftermath of events when available, could function as proxies for estimating how broad swaths of the population perceived both the event itself and leaderships’ response. It could also reveal opinion polling trends.

To create a chronological timeline, PubMed‐indexed studies describing the sequence of Covid‐19 events were utilized, as were Associated Press stories, White House Press Briefings, the David J. Spencer CDC Museum Covid‐19 timeline, and entries from Dr. Frieden's blog. Articles published from the beginning of the Covid‐19 pandemic through January 1, 2025, were reviewed. Perceived important occurrences were then cross‐referenced to ensure factuality and listed in a linear pattern. Public opinion polls and significant trending social media hashtags were included in the timeline to provide a comparative measure of public sentiment and online reaction.

In order to avoid selection bias, the primary author as well as a colleague–reviewer identified relevant events, announcements, social media trends, and polling data during that period. Disagreements concerning the significance or inclusion of an event, policy, announcement, or poll were resolved through the decision of a third colleague‐reviewer. For brevity, a 4000‐word limit was established to align with the typical constraints of most medical and health communication journals, while allowing space to be thorough. The SANRA checklist for narrative reviews was used as a reporting guideline.

To better understand the decline in public trust, two theoretical frameworks were incorporated to analyze the timeline of events: risk management theory and risk communication theory. Empirical research proposes that mistakes in evaluating, managing, or communicating risk to a population can lead to erosions of trust [4, 5]. Therefore, these frameworks were chosen because at the outset, the level of risk to the general public was uncertain but widely perceived to be high. Furthermore, effectively communicating this potential risk was considered essential, and doing so poorly could have adverse consequences.

## 3 Results

### 3.1 Literature Review

Forty‐seven sources, including research studies, news articles, public opinion and social media analyses, governmental documents, and systematic reviews, were identified and included in this study. Of these, 41 were used to establish the most significant events in the Covid‐19 pandemic timeline. The remaining six, representing influential publications on risk assessment, management, and communication theories, provided a framework for evaluating these events. Actions taken by pandemic‐era leaders and public health officials were then compared with established recommendations from risk assessment, management, and communication theorists to assess their concordance with, or divergence from, accepted principles. The results of this analysis are listed below.

### 3.2 Covid‐19 Timeline of Events

From December 2019 until summer 2020, the world witnessed events and a global public health response that had not occurred in over 100 years [6]. Cases of a severe respiratory illness were first reported in late 2019 in Wuhan, China, and the novel coronavirus that was subsequently identified spread internationally within weeks. The first confirmed case in the United States was announced on January 20, 2020 [6, 7, 8]. As transmission accelerated, the World Health Organization formally declared Covid‐19 a pandemic on March 11 [7]. During the same period, public health agencies introduced social distancing recommendations to reduce person‐to‐person transmission, including limits on gatherings and maintenance of physical separation in public spaces. By mid‐to‐late March 2020, multiple US states had implemented stay‐at‐home orders and widespread lockdown measures, resulting in school closures, business restrictions, and substantial disruptions to daily life [6, 9]. These initial key measures failed to consider Covello, Allen, and Fischhoff's recommendation that the public should be treated as a partner in decision‐making and that public concerns should be actively acknowledged and addressed [10, 11].

The summer of 2020 was notable for widespread protests across the United States following the death of George Floyd. This event marked one of the earliest examples of inconsistent public health messaging and leadership behavior. Until this period, nearly all prominent public health authorities, including Dr. Anthony Fauci, the Big Cities Health Coalition, former CDC Director Tom Frieden, and leaders of the nation's largest public health departments, had consistently emphasized the importance of remaining at home and minimizing interpersonal contact when possible, and maintaining a distance of at least 6 ft from others at all times when it was not [12]. However, following the onset of protests, over 1,200 physicians and public health professionals published an open letter endorsing the continuation of demonstrations, asserting that the pursuit of social justice outweighed the risks associated with potential viral transmission [13]. Although officials did not completely disregard public health concerns or the potential for disease dissemination, their position may have contributed to an increased risk of viral spread and heightened concern among medically vulnerable populations, thereby triggering the “morality” factor identified by Peter Sandman in his work on risk communication and public outrage [4]. Dr. Frieden and other leading public health advocates were among those vocally supporting this stance [14]. Additionally, various medical organizations participated in the protests while clad in white coats—an action that contrasted with their prior advisories urging home isolation [13, 14]. Notably, New York City's contact tracers were directed not to inquire whether individuals testing positive for Covid‐19 had attended the protests [15]. This decision diverged from established public health protocols and suggested to some observers that ideological objectives might be prioritized over transparency and disease containment [16]. Despite the efforts of healthcare professionals to communicate the complexity of the situation and competing dynamics, these conflicting public health messages violated the fifth principle of risk communication, which advises all spokespersons to deliver consistent directives [10].

Following the conclusion of protests, public health authorities resumed recommending against large public gatherings. In November 2020, Dr. Fauci advocated for travel restrictions, cautioned against holiday family visits, and urged officials to implement measures short of a full lockdown in anticipation of an impending increase in Covid‐19 cases [17].

By the end of 2020, Americans had endured a prolonged period without many customary social activities, including sporting events, religious services, conventions, and other gatherings. Prominent public health leaders continued to endorse the extension of containment measures. Dr. Frieden and several state officials criticized governors who refrained from imposing stay‐at‐home orders and travel limitations [18, 19]. Concurrently, many healthcare professionals recommended that schooling remain virtual, despite early data indicating that children represented the age group with the lowest Covid‐19 mortality rates [18, 20].

Subsequent analyses of the efficacy of containment measures revealed some were significantly more effective than others. Studies also showed that multiple measures, possibly in conjunction with the effects of the pandemic itself, produced unintended consequences [21, 22]. Perhaps the most profound impact was the widespread experience of social isolation from prolonged distancing and lockdowns that contributed to higher rates of depression, suicide, and mental health service utilization [23].

Regrettably, key US decision‐makers did not initially emphasize that public health recommendations were formulated on the basis of the best available evidence concerning a novel virus and that such guidance was subject to modification as new information emerged. Given that a global pandemic of this scale had not been encountered for over a century and that medical treatments and pharmaceuticals were limited at that time, the current circumstances were unprecedented. Due to this violation of the first stage of risk management and the fourth principle of risk communication, some within the medical community and the general public developed skepticism toward early policies [10, 11, 24, 25]. The phrase “we follow the science” became a common retort to critics, intended to encourage adherence to public health directives and silence detractors. However, due to limited data at that early stage and a multitude of unknowns, some interpreted this phrase as a directive to comply unquestioningly [26].

The January 6, 2021, incident at the US Capitol marked the commencement of the pandemic's second year and exposed further tensions in public health communication. Physicians and public health practitioners from Johns Hopkins, UCLA, Georgetown, and Boston Children's Hospital publicly condemned the event on national platforms. However, rather than focusing primarily on the illegality of such an unprecedented occurrence, their criticism centered on whether the gathering would engender massive viral transmission [27]. This unusual focus violated the risk management developmental stage of “acknowledging the public has accepted similar risks in the past”—notably during the summer protests of 2020 [11]. Interestingly, many of these experts had promoted participating in the nationwide demonstrations at that time, yet the potential viral transmission risks associated with those earlier mass gatherings received significantly less public scrutiny [27].

Throughout the pandemic, Covid‐19 messaging frequently appeared to be influenced by political considerations. Support for vaccination efforts fluctuated depending on the governing administration. For example, during a vice‐presidential debate, Kamala Harris expressed reluctance to receive a Covid‐19 vaccine produced during Donald Trump's presidency [28]. This sentiment was echoed by several prominent media figures [29]. However, after election to the vice‐presidency, she began publicly advocating for the same vaccine and completed her inoculation in early 2021 [28]. These examples of inconsistent guidance from public figures run counter to Hance, Chess, and Sandman's recommendation that risk communication avoid mixed messaging in order to maintain public trust [30]. Additionally, though vaccine development began during the Trump administration (Republican), polling data indicated vaccine hesitancy among Republicans increased after President Biden assumed office, without any changes in vaccine composition, manufacturing, or administration. This change was noted particularly among younger members of the party [31]. Furthermore, vaccine‐related policies, such as mandates and passports, tended to align with regional political affiliation, as many Republican‐led states prohibited such measures and Democratic‐led states enacted and enforced them [32].

In the midst of these politicized dynamics, the conduct of certain US leaders responsible for healthcare decisions likely contributed to public confusion and diminished trust. For example, President Trump's perplexing remarks during an April 2020 press briefing led some to believe he suggested imbibing disinfectants as a Covid‐19 treatment option [33]. Subsequently, Dr. Fauci was photographed at a baseball game with his mask lowered beneath his chin, despite prior advocacy for continuous mask use in public settings [34]. Speaker Nancy Pelosi was also observed in leaked footage, visiting a San Francisco salon without a mask in contravention of local mandates [35]. Over 2020 and 2021, several city mayors, senators, and governors were documented violating their own Covid‐19 restrictions [36]. These examples violated the fourth and fifth rules of risk communication and prompted public critique, frequently expressed on social media with the hashtag “do what I say, not what I do” [10, 37, 38]. Moreover, amid inconsistent guidelines and delays in vaccine data dissemination, CDC Director Rochelle Walensky publicly apologized for the agency's communication shortcomings [39].

Finally, the origin of SARS‐CoV‐2 remained a persistent and contentious issue throughout the pandemic. Early in the outbreak, Anthony Fauci publicly cited emerging scientific evidence suggesting that the virus most likely originated in bats and represented a natural zoonotic event [8, 40, 41]. He continued to endorse this explanation over subsequent years, characterizing the virus as having likely crossed from an animal host into humans [42]. However, it was later revealed during sworn congressional testimony and investigations that during a February 1, 2020, meeting with eleven other scientists, Dr. Fauci was informed of a potential laboratory origin for the virus [8, 43, 44, 45]. Multiple participants at that meeting voiced the need to quell this potential alternative theory and went on to write the influential paper, “The proximal origin of SARS‐CoV‐2,” that argued against a laboratory cause. It was also disclosed that Fauci provided uncredited editorial input on the manuscript prior to its submission [8, 40, 44, 45]. This failure to publicly acknowledge the existence of competing scientific hypotheses, while consistently promoting a single explanation, is difficult to reconcile with established risk communication principles emphasizing transparency, full disclosure of relevant information, responsiveness to public concerns, and the accurate communication of scientific uncertainty [4, 10, 11, 30].

## 4 Discussion

Over the course of the pandemic, nearly all of the seven rules of risk communication, the developmental stages of risk management, the guidelines for establishing trust and credibility, and the outrage factors of risk communication were violated at some point. Some were disregarded multiple times over.

These combined factors steadily contributed to a concerning erosion of trust in America's leaders and public health system en masse. According to a poll by *The Economist*, 28% of unvaccinated Americans cited “fear of side effects” as their principal reason for vaccine refusal, whereas nearly one‐quarter indicated “distrust of the government” as a key factor. Yet, distrust extends beyond the government to encompass public health officials and institutions more broadly. When surveyed about trust in specific individuals, Dr. Fauci received support from only 42% of respondents, Dr. Walensky garnered 19%, and the CDC and FDA each received roughly 50% [1]. Additional polling by the Robert Wood Johnson Foundation and Harvard Chan School of Public Health found that only 37% of Americans expressed strong trust in the NIH, with 48% indicating moderate or no trust. Approximately one‐third reported trusting the Department of Health and Human Services. Interestingly, whereas public trust in health agencies and leaders declined over this span, trust in physicians, nurses, and other frontline healthcare workers increased [1, 2].

Compounding the problems listed above, including reputational damage, the pandemic exacted a considerable emotional and physical toll on the public health workforce. By the end of 2021, more than 500 senior health officials had resigned or been removed from their positions—an event described as “the largest exodus of public health leaders in American history” [46].

From the lows described above, however, points of optimism remain. Confidence in US public health officials, governmental leaders, and health agencies can likely be restored—or at least improved—if these entities course correct and collectively make several simple changes. Instituting these changes should be easier than anticipated, given that we now have a blueprint for what not to do going forward. Any steps other than continuing in the currently charted course should be an improvement. First, future public health communications should be clear, concise, and easily comprehensible. In addition, these messages should be reinforced consistently. Insights from marketing and political campaign communications emphasize the importance of repetition and simplicity in effective messaging, as demonstrated by easily recalled advertising slogans and campaign mottos. Second, public health and governmental leaders must embrace transparency by disseminating available public health data early to demonstrate why certain policies are instituted. Third, leaders should engage the communities they serve to ensure that public health recommendations reflect the perspectives and desires of affected populations and receive broad public support. Fourth, leaders ought to issue prompt apologies when errors in health communication occur or less‐than‐helpful directives are issued. To this end, It is equally efficacious to communicate that guidance may evolve in response to emerging evidence, and the public should remain attentive to recommendation updates. Acknowledging the limits of current knowledge is acceptable and fosters trust. Finally, and perhaps of utmost importance, leaders should consistently adhere to their own policies. Advising one thing and doing another only engenders skepticism, criticism, and rebellion [4, 10, 30, 47].

## 5 Conclusion

The response of the US healthcare workforce to Covid‐19 was commendable, earning frontline workers deserved recognition as heroes. It is now incumbent upon health agencies and those in leadership to draw upon the successful communication strategies of other sectors in order to enhance public health messaging going forward. As society has moved into a post‐pandemic epoch, it is imperative that these entities and agents do so to restore trust, build a sense of camaraderie, and encourage healthy behaviors to improve and maintain the health of us all.

## Author Contributions

**Matthew L. Harrison**: conceptualization, investigation, writing – original draft, methodology, validation, visualization, writing – review and editing, formal analysis, project administration, supervision, resources.

## Funding

The author has nothing to report.

## Consent

This manuscript was prepared with waived informed consent by the UT Southwestern IRB.

## Conflicts of Interest

The author declares no conflicts of interest.

## Data Availability

All data are available from the author, upon request.

## References

[1] K. Frankovic , Why Aren't Some Americans Getting Vaccinated Against Covid‐19? Often, It's a Lack of Trust (YouGov, 2021).

[2] Harvard T.H. Chan School of Public Health ., The Public's Perspective on the United States Public Health System (Robert Wood Johnson Foundation, 2021), https://www.rwjf.org/en/insights/our‐research/2021/05/the‐publics‐perspective‐on‐the‐united‐states‐public‐health‐system.html.

[3] M. S. Pollard and L. M. Davis , “Decline in Trust in the Centers for Disease Control and Prevention During the COVID‐19 Pandemic,” RAND Health Quarterly 9, no. 3 (2022): 23, https://www.ncbi.nlm.nih.gov/pubmed/35837520. 10.7249/rhq-09-03-23 PMC9242572 35837520

[4] P. M. Sandman , “Risk Communication: Facing Public Outrage,” EPA Journal 2, no. 2 (November 1987): 21–22, https://www.psandman.com/articles/facing.htm.

[5] National Academy of Sciences , Improving Risk Communication (National Academy Press, 1989).

[6] D. M. Morens , J. K. Taubenberger , and A. S. Fauci , “A Centenary Tale of Two Pandemics: The 1918 Influenza Pandemic and COVID‐19, Part II,” American Journal of Public Health 111, no. 7 (2021): 1267–1272, 10.2105/AJPH.2021.306326. 34111372 PMC8493155

[7] T. Carvalho , F. Krammer , and A. Iwasaki , “The First 12 Months of COVID‐19: A Timeline of Immunological Insights,” Nature Reviews Immunology 21, no. 4 (2021): 245–256, 10.1038/s41577-021-00522-1. PMC7958099 33723416

[8] T. W. House , “Lab Leak: The True Origins of Covid‐19,” published (The White House, 2024), https://www.whitehouse.gov/lab‐leak‐true‐origins‐of‐covid‐19/.

[9] Z. Allam , Surveying the Covid‐19 Pandemic and Its Implications: Urban Health, Data Technology and Political Economy (Elsevier, 2020).

[10] V. T. Covello , “Decision Analysis and Risk Management Decision Making: Issues and Methods,” Risk Analysis 7, no. 2 (1987): 131–139, 10.1111/j.1539-6924.1987.tb00978.x. 3112872

[11] B. Fischhoff , “Risk Perception and Communication Unplugged: Twenty Years of Process,” Risk Analysis 15, no. 2 (1995): 137–145, 10.1111/j.1539-6924.1995.tb00308.x. 7597253

[12] M. Mays , Nearly 30 of the Nation's Largest Public Health Departments Launch Ad Campaign Urging People to Stay Home (County News Center, 2020), https://www.bigcitieshealth.org/newsroom‐publichealth‐leaders‐openletter/.

[13] T. Haelle , Risking Their Lives to Save Their Lives: Why Public Health Experts Support Black Lives Matter Protests (Forbes, 2020), https://www.forbes.com/sites/tarahaelle/2020/06/19/risking‐their‐lives‐to‐save‐their‐lives‐why‐public‐health‐experts‐support‐black‐lives‐matter‐protests/?sh=18cd545a851b.

[14] R. Weiner , “Political and Health Leaders' Embrace of Floyd Protests Fuels Debate Over Coronavirus Restrictions,” Washington Post June 11, 2020, https://www.washingtonpost.com/health/political‐and‐health‐leaders‐embrace‐of‐floyd‐protests‐fuels‐debate‐over‐coronavirus‐restrictions/2020/06/11/9c60bca6‐a761‐11ea‐bb20‐ebf0921f3bbd_story.html.

[15] I. Scher , “NYC's Contact Tracers Have Been Told Not to Ask People If They've Attended a Protest,” Business Insider June 16, 2020, https://www.businessinsider.com/nyc‐contact‐tracers‐not‐asking‐people‐attend‐george‐floyd‐protest‐2020‐6.

[16] L. Emmons , “New York City Mayor Tells Contact Tracers to Not Ask If Covid Positive People Attended George Floyd Protests,” Post Millenial June 15, 2020, https://thepostmillennial.com/new‐york‐city‐mayor‐tells‐contact‐tracers‐to‐not‐ask‐if‐covid‐positive‐people‐attended‐george‐floyd‐protests.

[17] E. Newburger , Fauci Says Christmas and New Year's Restrictions Will Be Necessary Due to Holiday Coronavirus Wave (CNBC, 2020), https://www.cnbc.com/2020/11/29/coronavirus‐fauci‐says‐christmas‐and‐new‐years‐restrictions‐will‐be‐necessary.html.

[18] T. Frieden , “Covid Epi Weekly,” published (Substack, 2020), http://drtomfrieden.net/blog/.

[19] B. Melley , “West Coast Governors Urge COVID Quarantine After Travel,” AP News October 15, 2020, https://apnews.com/article/travel‐kate‐brown‐washington‐coronavirus‐pandemic‐oregon‐343b631667a6f67bfc9e8aae22e0e24d.

[20] UNICEF , Child Mortality and Covid‐19 (UNICEF, 2021), https://data.unicef.org/topic/child‐survival/covid‐19/.

[21] T. Jefferson , L. Dooley , E. Ferroni , et al., “Physical Interventions to Interrupt or Reduce the Spread of Respiratory Viruses,” Cochrane Database of Systematic Reviews 1, no. 1 (2023): CD006207, 10.1002/14651858.CD006207.pub6. 36715243 PMC9885521

[22] B. Nussbaumer‐Streit , V. Mayr , A. I. Dobrescu , et al., “Quarantine Alone or in Combination With Other Public Health Measures to Control COVID‐19: A Rapid Review,” Cochrane Database of Systematic Reviews 9, no. 9 (2020): CD013574, 10.1002/14651858.CD013574.pub2. 33959956 PMC8133397

[23] M. Ernst , D. Niederer , A. M. Werner , et al., “Loneliness Before and During the Covid‐19 Pandemic: A Systematic Review With Meta‐Analysis,” American Psychologist 77, no. 5 (2022): 660–677, 10.1037/amp0001005. 35533109 PMC9768682

[24] C. Chaufan and N. Hemsing , “Is Resistance to Covid‐19 Vaccination a “Problem”? A Critical Policy Inquiry of Vaccine Mandates for Healthcare Workers,” AIMS Public Health 11, no. 3 (2024): 688–714, 10.3934/publichealth.2024035. 39416898 PMC11474332

[25] M. Motta and T. Callaghan , “The 1 in 10 U.S. Doctors With Reservations About Vaccines Could be Undermining the Fight Against Covid‐19,” Texas A&M Today April 5, 2022, https://today.tamu.edu/news/2022/04/05/the-1-in-10-u-s-doctors-with-reservations-about-vaccines-could-be-undermining-the-fight-against-covid-19/.

[26] A. Berezow , Coronavirus: ‘Follow the Science’ Means ‘Do What I Say’ (American Council on Science and Health, 2020), https://www.acsh.org/news/2020/04/28/coronavirus‐follow‐science‐means‐do‐what‐i‐say‐14748.

[27] A. Mitropoulos , “Capitol Hill Riot Could Prove to be Covid‐19 Superspreader Event, Experts Say,” ABC News January 9, 2021), https://abcnews.go.com/Health/capitol‐hill‐riot‐prove‐covid‐19‐superspreader‐event/story?id=75134968.

[28] K. Doyle , Kamala Harris Flips From Vaccine Skeptic to Vaccine Booster (Washington Examiner, 2021), https://vip-stage.washingtonexaminer.com/news/2878352/kamala-harris-flips-from-vaccine-skeptic-to-vaccine-booster/.

[29] J. Lancaster , “Analysis: The Left Said It Wouldn't Trust the Trump Vaccine. Now They're Shaming the Unvaccinated,” Daily Caller July 30, 2021, https://dailycaller.com/2021/07/30/public-figures-trust-trump-vaccine-shaming-unvaccinated-kamala-harris/.

[30] B. J. Hance , C. Chess , and P. M. Sandman , Improving Dialogue With Communities: A Risk Communication Manual for Government: Submitted to New Jersey Department of Environmental Protection, Division of Science & Research (The Division, 1988).

[31] Y. Alcindor , “Why 41 Percent of Republicans Don't Plan to Get the Covid Vaccine,” PBS News Hour March 19, 2021, https://www.pbs.org/newshour/show/why‐41‐percent‐of‐republicans‐dont‐plan‐to‐get‐the‐covid‐vaccine.

[32] J. Drees , Vaccine Passports: 50 States With Bans, Limitations & Greenlights (Becker's Clinical Leadership, 2021), https://www.beckershospitalreview.com/digital‐transformation/vaccine‐passports‐10‐states‐with‐bans‐limitations‐green‐lights.html.

[33] J. Jarvis , “Fact Check: Did Donald Trump Suggest People Inject Poison to Cure COVID?” Newsweek August 16, 2021, https://www.newsweek.com/fact‐check‐did‐donald‐trump‐suggest‐people‐inject‐poison‐cure‐covid‐1619105.

[34] A. Swoyer , “Dr. Anthony Fauci Says Photo of Him Without a Mask at Baseball Game Is ‘Mischievous’,” Washington Times July 24, 2020, https://www.washingtontimes.com/news/2020/jul/24/dr‐anthony‐fauci‐says‐photo‐him‐without‐mask‐baseb/.

[35] T. Barrett , “Pelosi's Office Acknowledges Indoor Hair Appointment, Violating San Francisco Covid‐19 Restrictions,” CNN Politics September 3, 2020, https://www.cnn.com/2020/09/02/politics/nancy‐pelosi‐hair‐salon/index.html.

[36] G. Panetta , “14 Prominent Democrats Stand Accused of Hypocrisy for Ignoring Covid‐19 Restrictions They're Urging Their Constituents to Obey,” Business Insider September 21, 2021, https://www.businessinsider.com/democratic‐politicians‐who‐violated‐covid‐19‐rules‐guidance‐list‐2020‐12?op=1.

[37] P. E. Board , “Democrat Hypocrites Are Undermining Covid With “Do as I Say, Not as I Do” Attitude,” *New York Post*: December 3, 2020: 1, 4, 5, https://nypost.com/2020/12/03/democrats‐undermining‐covid‐with‐do‐as‐i‐say‐not‐as‐i‐do/.

[38] M. Deliso , “Elected Officials Slammed for Hypocrisy for Not Following Own Covid‐19 Advice,” ABC News December 4, 2020, https://abcnews.go.com/Health/elected‐officials‐criticized‐covid‐19‐advice/story?id=74486861.

[39] S. Toy , “CDC Director Aims to Improve Covid‐19 Messaging, Data Collection,” Wall Street Journal January 17, 2022, https://www.wsj.com/articles/cdc‐director‐aims‐to‐improve‐covid‐19‐messaging‐data‐collection‐11642429801.

[40] K. G. Andersen , A. Rambaut , W. I. Lipkin , E. C. Holmes , and R. F. Garry , “The Proximal Origin of SARS‐CoV‐2,” Nature Medicine 26, no. 4 (2020): 450–452, 10.1038/s41591-020-0820-9. PMC7095063 32284615

[41] “Remarks by President Trump, Vice President Pence, and Members of the Coronavirus Task Force in Press Briefing,” White House Archives, published 2020, https://trumpwhitehouse.archives.gov/briefings‐statements/remarks‐president‐trump‐vice‐president‐pence‐members‐coronavirus‐task‐force‐press‐briefing‐april‐17‐2020/.

[42] A. Fauci , Coronavirus Likely Caused by Natural Occurrence Rather Than Lab Leak, Dr. Fauci Says (C‐SPAN, 2022), https://www.c‐span.org/clip/senate‐committee/coronavirus‐likely‐caused‐by‐natural‐occurrence‐rather‐than‐lab‐leak‐dr‐fauci‐says/5020088.

[43] J. Comer , Fauci's Emails Raise Questions of Lab Leak Cover Up (Congress of the United States, 2022), https://comer.house.gov/2022/1/comer‐fauci‐s‐emails‐raise‐questions‐of‐lab‐leak‐cover‐up.

[44] R. J. Comer , Letter to Xavier Becerra—Secretary of the U.S. Dept of HHS (Committee on Oversight and Reform, published 2022), https://oversight.house.gov/wp‐content/uploads/2022/01/Letter‐Re.‐Feb‐1‐Emails‐011122.pdf.

[45] Congress of the United States , *Select Subcommittee on the Coronavirus Pandemic Member Memorandum: New Evidence Resulting From the Select Subcommittee's Investigation Into the Origins of COVID‐19*—“*T*he Proximal Origin of SARS‐CoV‐2” (Congress of the United States, 2023), https://oversight.house.gov/wp‐content/uploads/2023/03/2023.03.05‐SSCP‐Memo‐Re.‐New‐Evidence.Proximal‐Origin.pdf.

[46] J. K. Varma , T. G. Long , and D. A. Chokshi , “5 Skills Public Health Officials Need to Combat the next Pandemic,” in Harvard Business Review, Crisis Management. (Harvard Business Review Press, 2021), https://hbr.org/2021/12/5‐skills‐public‐health‐officials‐need‐to‐combat‐the‐next‐pandemic.

[47] R. Schiavo , Health Communication: From Theory to Practice, 2nd ed. (Wiley Publishers, 2014).

